# Analgesic effect of the aqueous and ethanolic extracts of clove

**Published:** 2013

**Authors:** Mina Kamkar Asl, Ashraf Nazariborun, Mahmoud Hosseini

**Affiliations:** 1 *Pharmacological Research Center of Medicinal Plants, School of Medicine, Mashhad University of Medical Sciences, Mashhad, I. R. Iran*; 2 *Department of Physiology, School of Medicine, Zabol University of Medical Sciences, Zabol, I. R. Iran*; 3 *Neuroscience Research Center and Department of Physiology, School of Medicine, Mashhad University of Medical Sciences, Mashhad I. R. Iran*

**Keywords:** Analgesia, Clove, Extract, Hot Plate, Mice, Naloxone

## Abstract

**Objective: **The beneficial effects of clove on toothache have been well documented. We have also previously shown the analgesic effects of clove essential oil. The present work was done to investigate the analgesic effects of the aqueous extract of clove using hot plate test. The possible role of opioid receptors in the analgesic effects of clove was also investigated using naloxone.

**Materials and Methods**: Ninety male mice were divided into nine groups: (1) Saline, (2-4) Aaqueous (Aq 50, Aq 100, and Aq 200) groups which were treated with 50, 100, and 200 mg/kg of aqueous extract of clove, respectively, (5-7) Ethanolic (Eth 50, Eth 100, and Eth 200) groups which were treated with 50, 100, and 200 mg/kg of ethanolic extract of clove, respectively, and (8-9) Aq 100- Naloxone and Aq 200- Naloxone which were pretreated with 4 mg/kg of naloxone before injection of 100 or 200 mg/kg of the aqueous extract. The hot plate test was performed as a base record 10 min before injection of drugs and consequently repeated every 10 minutes after the injection.

**Results: **The maximal percent effect (MPE) in the animal groups treated with 50, 100, and 200 mg/kg of aqueous extract was significantly higher than the control group. Pretreatment with naloxone reduced the analgesic effects of both 100 and 200 mg/kg of the aqueous extract. Administration of all three doses of the ethanloic extract also non-significantly increased the MPE.

**Conclusion: **The results of the present study showed that aqueous extract of clove has analgesic effect in mice demonstrated by hot plate test which is reversible by naloxone. The role of opioid system in the analgesic effect of clove might be suggested. However, more investigations are needed to elucidate the exact mechanism(s).

## Introduction

 The clove* (Eugenia caryophyllata*) is a tree with 10 to 20 meters in height from Myrtaceae family which is cultivated in some countries such as Malaysia, Indonesia, Sri Lanka, Madagascar, India, and Tanzania (Arung et al., 2011 ▶). Its parts including leaves and buds are commercially used in cooking, food processing, and perfumery (Daniel et al., 2009 ▶). Some parts of the plant have been shown to be useful for treating the digestive system disorders (Baytop, 1999 ▶). Some components of clove have been advised against bacterial and fungal infections (Zhang and Chen, 1997 ▶; Zheng et al., 1992 ▶). It has also been documented that some parts of the plant and its ingredients have a good cytotoxic and even anti-cancerogenic properties (Kouidhi et al., 2010 ▶; Zhang and Chen, 1997 ▶; Zheng et al., 1992 ▶). The extracts of this plant have been considered to have benefits against oral bacteria especially those which are accompanied with dental caries and periodontal diseases (Cai and Wu, 1996 ▶). The clove oil has also been used for acne, warts, scars, and parasites (Saeed and Tariq, 2008 ▶). It has also been shown that the essential oil form clove inhibits the smooth muscle tone (Damiani et al., 2003 ▶; Nishijima et al., 1999 ▶). The useful effects of the plant in allergic asthma have also been reported (Kim et al., 1998 ▶).

 The analgesic effects of the plant in subjects suffering from toothache and anal fissure have been reported (Elwakeel et al., 2007 ▶). The anesthetic effects of the essential oil from several parts of this plant have also been shown in fish (^Park et al., 2011a ▶^). Using animal models, the anesthetic effects of eugenol, the main component of clove, as well as its analgesic and anti-inflammatory effects have been well documented (Daniel et al., 2009 ▶; Diaz and Sembrano, 1985 ▶; Kurian et al., 2006 ▶; Oztürk and Ozbek, 2005 ▶; Yu and Hungju, 1981 ▶). Pharmacological studies have also demonstrated the anticonvulsant and anti-stress properties of eugenol (Dallmeier and Carlini, 1981 ▶; Sen et al., 1992 ▶). In traditional medicine, the buds of this plant have been used as an antiepileptic remedy (Avicenna, 1988 ▶). We have also previously shown the analgesic effects of clove essential oil (Hosseini et al., 2011 ▶). Therefore, the present work was undertaken in order to investigate the possible analgesic effects of ethanolic and aqueous extracts of clove in mice. Furthermore, the role of opioid system in analgesic effects of the aqueous extract of clove was examined using naloxone.

## Material and Methods

**Animals and drugs**

 Ninety BALB/c male mice (27-32 g) were used. All mice were housed in 4–6 per standard cage at room temperature 23±1 °C) on a 12 h light/dark cycle. Food and water were available ad libitum. Animal handling and all related procedures were carried out in accordance with Mashhad University of Medical Sciences, Ethical Committee Acts. The clove was kindly provided by Eksir Gol Sorkh Company, Mashhad, Iran. 

The chopped, dried buds were extracted using a Soxhlet apparatus with 300 ml distilled water and ethanol to prepare aqueous and ethanolic extracts, respectively. The extracts reduced to dryness with a rotary vacuum evaporator, yielded 15% and 17%, for aqueous and ethanolic extracts, respectively.

**Nociceptive test**

 To assess nociceptive responses, hot plate method was used. In hot plate method, animals were placed on the hot plate with temperature setting controlled at 55±0.2 °C. Cut-off time was 60 seconds (Karami et al., 2011 ▶). Time duration between placing the animals on hot plate and licking forepaws or moving hind paws was considered as reaction time. The hot plate test was performed as a three base record before injection of the extract or vehicle and consequently was repeated 5 times, every 10 minutes after the injection (Hosseini et al., 2009 ▶; Karami et al., 2011 ▶; Rakhshandeh et al., 2008 ▶). 

**Experimental design**

Ninety mice were divided into nine groups (n=10 in each): (1) Control: the animals of this group received vehicle, (2-4) Aqueous extract groups (Aq 50, Aq 100, and Aq 200) which were treated with 50, 100, and 200 mg/kg of aqueous extract of clove, respectively, (5-7) Ethanolic extract groups (Eth 50, Eth 100, and Eth 200) which were treated with 50, 100, and 200 mg/kg of ethanolic extract of clove, respectively, and (8-9) Aqueous extract groups pretreated by Naloxone (Nlx) (Aq 100- Nlx and Aq 200- Nlx) which were pretreated with 4 mg/kg of naloxone (Rakhshandeh et al., 2008) before injection of 100 or 200 mg/kg of the aqueous extract. All drugs were injected intraperitonealy (i.p.) in a volume of 10 ml/kg. 

**Statistical analysis**

Analgesic effects of the extracts or vehicle were calculated as maximal possible effect (MPE) [MPE (%) = [(test response time-basal response time)/(cut-off time-basal response time)×100%] (Sepehri and Shafeiee, 2006). All data were presented as mean±SEM of %MPE. Repeated measures ANOVA followed by post hoc Tukey^’^s test was used for comparison of %MPE after injection of drugs. Differences were considered statistically significant when p<0.05

## Results

**Figure 1:**
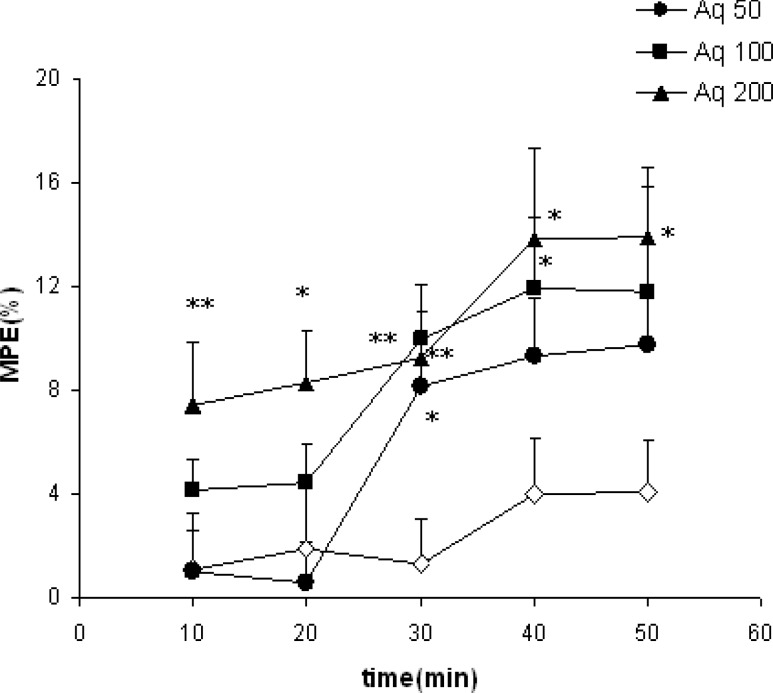
Comparison of MPE between the groups which received different doses of aqueous extract or vehicle. Data were shown as mean±SEM (n=10 in each group).^ *^P<0.05, ^**^P<0.01 compared with the control group. Aq 50: Aqueous extract 50, Aq 100: Aqueous extract 100 and Aq 200: Aqueous extract 200. Repeated measures ANOVA followed by post hoc Tukey^’^s test was used for comparison of %MPE after injection of drugs

MPE in animal groups treated with 50 mg/kg of the aqueous extract of clove was significantly higher than the vehicle-treated animals at 30 minutes after injection (p<0.05, Figure 1).

Treatment of the animals by 100 mg/kg of the aqueous extract of clove significantly increased the MPE at 30 and 40 minutes after the injection (p<0.01 and p<0.05, respectively, Figure 1). Figure 1 also shows that the animals of Aq 200 group had more MPE at all times after injection compared with the control group (p<0.05 - p<0.01).

**Figure 2:**
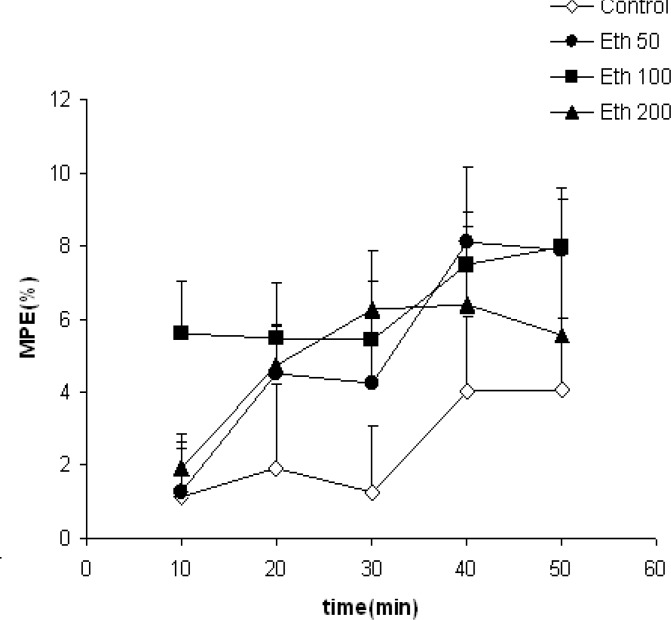
Comparison of MPE between the groups which received different doses of ethanolic extract or vehicle. Data were shown as mean±SEM (n=10 in each group). There was not significant differences in the effect of ethanolic extract compared with the control group. Eth 50: Ethanolic extract 50, Eth 100: Ethanolic extract 100 and Eth 200: Ethanolic extract 200. Repeated measures ANOVA followed by post hoc Tukey^’^s test was used for comparison of %MPE after injection of drugs

As the Figure 2 shows, the MPE in animal groups treated with 50-200 mg/kg of the ethanolic extract of clove was higher than the vehicle-treated animals. However, the differences were not significant.

**Figure 3:**
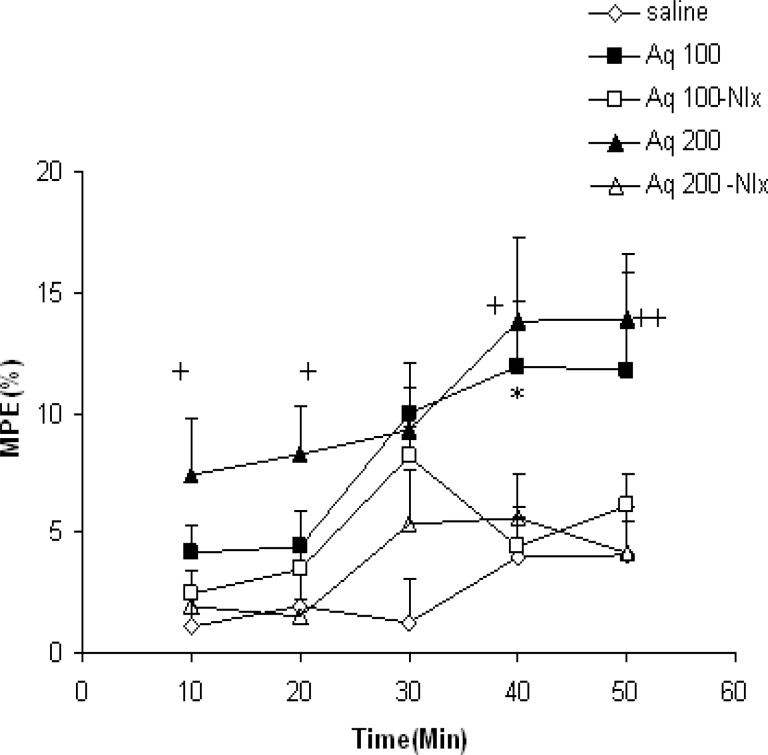
Comparison of MPE between the groups which received 100 or 200 mg/kg of aqueous extract and the groups which were pretreated with naloxone before injection of the extract. Data were shown as mean±SEM (n=10 in each group). ^*^p<0.05 compared with Aq 100- Nlx. ^+^p<0.05, ^++^p<0.01 compared with Aq 200- Nlx. Aq 100- Nlx: Aqueous extract 100- Naloxone and Aq 200- Nlx: Aqueous extract 200- Naloxone. Repeated measures ANOVA followed by post hoc Tukey^’^s test was used for comparison of %MPE after injection of drugs

In the present study, the effects of pretreatment with naloxone on the analgesic effects of 100 and 200 mg/kg of the extract were also examined. As the results show, the MPE in Aq 100- Nlx group was lower than the Aq 100 group at 40 minutes after the injection (p<0.05, Figure 3). The results also showed that pretreatment with naloxone reduced the MPE at 10, 20, 40, and 50 minutes after injection of 200 mg/kg of the aqueous extract (p<0.05 - p<0.01, Figure 3).

## Discussion

In the present study, it was demonstrated that aqueous but not ethanolic extract from clove had analgesic effects tested in hot plate. A maximum effect of 200 mg/kg of the aqueous extract was seen 50 minutes after the injection. To obtain better insight into the nature of compounds responsible for the analgesic effect of clove, two extracts were prepared: (1) The aqueous extract which contain polar constituents and (2) The ethanolic extract which bears the medium- to low-polarity compounds (Seidel, 2006 ▶). Our observation that ethanolic extract did not significantly affect MPE as an analgesia parameter indicates that medium-polarity and lipophilic compounds (e.g. alkalenes, fatty acids, and steroles) are not responsible for the analgesic effect of clove. Therefore, it is reasonable to assume that polar constituents of clove are responsible for the anticonvulsive activity. However, there are some reports that eugenol, an aromatic molecule derived from essential oil of clove, exerts the analgesic activity (Muller et al., 2006 ▶). 

It has been well documented that different parts of clove are very useful in toothache (Yu and Hungju, 1981 ▶). The anesthetic properties of the extracts of this plant have also been frequently reported in fish which was comparable with lidocaine (Anderson et al., 1997 ▶; Waterstrat, 1999 ▶; Park et al., 2011a ▶). The topical application of clove oil cream has been shown to have a beneficial effect in patients suffering from chronic anal fissure (Elwakeel et al., 2007 ▶). Several components have been reported for clove. The highest concentrations are due to eugenol (88.58%), eugenyl acetate (5.62%), and β-cariophyllene (1.38%) (Chaieb et al., 2007 ▶; Oztürk and Ozbek, 2005 ▶). The analgesic effect of clove which was seen in the present study may be due to eugenol. Using the chemical (acetic acid tests), as well thermal methods, the antinociceptive activity of eugenol has been well documented (Daniel et al., 2009 ▶; Kurian et al., 2006 ▶). The analgesic effect of eugenol has been attributed to its capability for inhibiting the prostaglandins and other inflammatory mediators such as leukotrienes (Raghavenra et al., 2006 ▶). Anti-inflammatory, antipyretic, and anti allergic effects of this compound may also be other explanations for this idea (Feng and Lipton, 1987 ▶; Murakami et al., 2003 ▶). Eugenol also reduces the paw edema and the pleural exudates in carrageenan-induced inflammation model (Daniel et al., 2009 ▶). Eugenol is also believed to block the pain receptors (Halder et al, 2012). 

Considering the results of the present study, it seems that the aqueous extract of clove has analgesic effects with mechanism(s) which differ from previous studies. Eugenol also blocks the conduction of action potential in sciatic nerves (Kozam, 1977 ▶). Eugenol induces an anesthesia in rodents which is comparable with propofol (Guénette et al., 2007 ▶; Sell and Carlini, 1976 ▶). It also alleviates the thermal hyperalgesia in an animal model of neuropathic pain (Guénette et al., 2007 ▶). Eugenol inhibits *N*-methyl-d-aspartate (NMDA) receptors which are involved in pain sensitivity (Aoshima and Hamamoto, 1999 ▶). Eugenol is similar in chemical structure to capsaicin and therefore its effect on a vanilloid receptor should not be ignored (Yang et al., 2003 ▶). It was also shown that eugenol inhibits Na^+^ currents in rat dorsal root ganglion neurons (Cho et al., 2008 ▶). β-Caryophyllene, the other main component of clove oil, showed anti-inflammatory activity in several animal models, including carrageenan- and prostaglandin E -induced hind paw edema (Ghelardini et al., 2001 ▶)**.** The role of this component in analgesic effects of clove which was shown in the present study should not be ignored. 

The results of the present study also showed that naloxone attenuated the analgesic effects of the aqueous extract. Therefore, the involvement of opioid system in the analgesic effects of clove might be suggested. It has also been previously reported that eugenol potentiates ionotropic γ-aminobutyric acid (GABA_A_) receptors (Aoshima and Hamamoto, 1999 ▶). It has also been reported that orally administration of eugenol has analgesic effects which is reversible by naloxone (Park et al., 2011b ▶; Paula-Freire et al., 2012 ▶). 

In conclusion, regarding the results of the present study, it might be suggested that besides eugenol, there are also other polar compounds in buds of clove which have analgesic effects, but it needs to be further investigated. It is also concluded that aqueous extract of clove have analgesic effects which may at least in part be due to opioid system. However, the exact mechanism(s) need to be investigated.

## Conﬂict of interest

The authors declare that there are no conﬂicts of interest.

## References

[B1] Anderson WG, McKinley RS, Colavecchia M (1997). The use of clove oil as an anesthetic for rainbow trout and its effects on swimming performance. N Am J Fish Manag.

[B2] Aoshima H, Hamamoto K (1999). Potentiation of GABAA receptors expressed in Xenopus oocytes by perfume and phytoncid. Biosci Biotechnol Biochem.

[B3] Arung ET, Matsubara E, Kusuma IW, Sukaton E, Shimizu K, Kondo R (2011). Inhibitory components from the buds of clove (Syzygium aromaticum) on melanin formation in B16 melanoma cells. Fitoterapia.

[B4] Avicenna A (1988). Ghanoon dar Teb.

[B5] Baytop T (1999).

[B6] Cai L, Wu CD (1996). Compounds from Syzygium aromaticum possessing growth inhibitory activity against oral pathogens. J Nat Prod.

[B7] Chaieb K, Hajlaoui H, Zmantar T, Kahla Nakbi AB, Rouabhia M, Mahdouani K, Bakhrouf A (2007). The chemical composition and biological activity of clove essential oil, Eugenia caryophyllata (Syzigium aromaticum L. Myrtaceae): a short review. Phytother Res.

[B8] Cho JS, Kim TH, Lim JM, Song JH (2008). Effects of eugenol on Na+ currents in rat dorsal root ganglion neurons. Brain Res.

[B9] Dallmeier K, Carlini EA (1981). Anesthetic, hypothermic, myorelaxant and anticonvulsant effects of synthetic eugenol derivatives and natural analogues. Pharmacol Pharmacol.

[B10] Damiani CEN, Rossoni LV, Vassallo DV (2003). Vasorelaxant effects of eugenol on rat thoracic aorta. Vasc pharmacol.

[B11] Daniel AN, Sartoretto SM, Schmidt G, Caparroz-Assef SM, Bersani-Amado CA, Cuman R KN (2009). Anti-inflammatory and antinociceptive activities A of eugenol essential oil in experimental animal models. Rev Bras Farmacogn.

[B12] Diaz MR, Sembrano JM (1985). A comparative study of the efficacy of garlic and eugenol as palliative agents against dental pain of pulpal origin. J Philipp Dent Assoc.

[B13] Elwakeel HA, Moneim HA, Farid M, Gohar AA (2007). Clove oil cream: a new effective treatment for chronic anal fissure. Colorectal Dis.

[B14] Feng J, Lipton JM (1987). Eugenol: Antipyretic activity in rabbits. Neuropharmacol.

[B15] Ghelardini C, Galeotti N, Di Cesare Mannelli L, Mazzanti G, Bartolini A (2001). Local anaesthetic activity of [beta]-caryophyllene. Il Farmaco.

[B16] Guénette SA, Ross A, Marier JF, Beaudry F, Vachon P (2007). Pharmacokinetics of eugenol and its effects on thermal hypersensitivity in rats. Eur J pharmacol.

[B17] Halder S, Mehta AK, Mediratta PK, Sharma KK.2012 (Acute effect of essential oil of Eugenia caryophyllata on cognition and pain in mice). Naunyn Schmiedebergs Arch Pharmacol.

[B18] Hosseini M, Asl MK, Rakhshandeh H (2011). Analgesic effect of clove essential oil in mice. Avicenna J Phytomed.

[B19] Hosseini M, Sadeghnia HR, Salehabadi S, Alavi H, Gorji A (2009). The effect of L-arginine and L-NAME on pentylenetetrazole induced seizures in ovariectomized rats, an in vivo study. Seizure.

[B20] Karami R, Hosseini M, Khodabandehloo F, Khatami L, Taiarani Z (2011). Different effects of L-arginine on morphine tolerance in sham and ovariectomized female mice. J Zhejiang Univ Sci B.

[B21] Kim HM, Lee EH, Hong SH, Song HJ, Shin MK, Kim SH, Shin TY (1998). Effect of Syzygium aromaticum extract on immediate hypersensitivity in rats. J ethnopharmacol.

[B22] Kouidhi B, Zmantar T, Bakhrouf A (2010). Anticariogenic and cytotoxic activity of clove essential oil (Eugenia caryophyllata) against a large number of oral pathogens. Ann Microbiol.

[B23] Kozam G (1977). The effect of eugenol on nerve transmission. Oral Surg Oral Med Oral Pathol.

[B24] Kurian R, Arulmozhi DK, Veeranjaneyulu A, Bodhankar SL (2006). Effect of eugenol on animal models of nociception. Indian J of Pharmacol.

[B25] Muller M, Pape HC, Speckmann EJ, Gorji A (2006). Effect of eugenol on spreading depression and epileptiform discharges in rat neocortical and hippocampal tissues. Neurosci.

[B26] Murakami Y, Shoji M, Hanazawa S, Tanaka S, Fujisawa S (2003). Preventive effect of bis-eugenol, a eugenol ortho dimer, on lipopolysaccharide-stimulated nuclear factor kappa B activation and inflammatory cytokine expression in macrophages. Biochem pharmacol.

[B27] Nishijima H, Uchida R, Kameyama K, Kawakami N, Ohkubo T, Kitamura K (1999). Mechanisms mediating the vasorelaxing action of eugenol, a pungent oil, on rabbit arterial tissue. Jpn J Pharmacol.

[B28] Oztürk A, Ozbek H (2005). The anti-inflammatory activity of Eugenia caryophyllata essential oil: an animal model of anti-inflammatory activity. Eur J Gen Med.

[B29] Park IS, Park SJ, Gil HW, Nam YK, Kim DS (2011a). Anesthetic effects of clove oil and lidocaine-HCl on marine medaka (Oryzias dancena). Lab animal.

[B30] Park SH, Sim YB, Lee JK, Kim SM, Kang YJ, Jung JS, Suh H (W.2011b). The analgesic effects and mechanisms of orally administered eugenol. Arch Pharm Res.

[B31] Paula-Freire LI, Andersen ML, Molska GR, Kohn DO, Carlini EL (2012). Evaluation of the Antinociceptive Activity of Ocimum gratissimum L. (Lamiaceae) Essential Oil and Its isolated Active Principles in Mice. Phytother Res.

[B32] Raghavenra H, Diwakr BT, Lokesh BR, Naidu KA (2006). Eugenol--The active principle from cloves inhibits 5-lipoxygenase activity and leukotriene-C4 in human PMNL cells. Prostaglandin Leukot Essent Fatty Acids.

[B33] Rakhshandeh H, Vahdati-Mashhadian N, Dolati K, Hosseini M (2008). Antinociceptive effect of Rosa damascena in Mice. J Biol Sci.

[B34] Saeed S, Tariq P (2008). In vitro antibacterial activity of clove against Gram negative bacteria. Pakistan J Botany.

[B35] Seidel 2006.

[B36] Sell AB, Carlini EA (1976). Anesthetic action of methyleugenol and other eugenol derivatives. Pharmacology.

[B37] Sen P, Maiti PC, Puri S, Ray A, Audulov NA, Valdman AV (1992). Mechanism of anti-stress activity of Ocimum sanctum Linn, eugenol and Tinospora malabarica in experimental animals. Indian J experiment boil.

[B38] Sepehri G, Shafeiee MN (2006). Effect of Cuneiformis Nucleus Inactivation by Lidocaine Microinjection on the Analgesic Response of Morphine in Rats. Iran Biomed J.

[B39] Waterstrat PR (1999). Induction and recovery from anesthesia in channel catfish Ictalurus punctatus fingerlings exposed to clove oil. J World Aquacult Soc.

[B40] Yang BH, Piao ZG, Kim YB, Lee CH, Lee JK, Park K, Kim JS, Oh SB (2003). Activation of vanilloid receptor 1 (VR1) by eugenol. J Dent Res.

[B41] Yu J, Hungju F (1981). Studies on the essential oils of clove buds and clove leaves. Zhong Caoyao.

[B42] Zhang Y, Chen Y (1997). Isobiflorin, a chromone C-glucoside from cloves (Eugenia caryophyllata). Phytochemistry.

[B43] Zheng GQ, Kenney PM, Lam LKT (1992). Sesquiterpenes from clove (Eugenia caryophyllata) as potential anticarcinogenic agents. J Nat Prod.

